# The Ratio of Hmox1/Nrf2 mRNA Level in the Tumor Tissue Is a Predictor of Distant Metastasis in Colorectal Cancer

**DOI:** 10.1155/2016/8143465

**Published:** 2016-11-23

**Authors:** Liang-Che Chang, Chung-Wei Fan, Wen-Ko Tseng, Hui-Ping Chein, Tsan-Yu Hsieh, Jim-Ray Chen, Cheng-Cheng Hwang, Chung-Ching Hua

**Affiliations:** ^1^Department of Pathology, Chang Gung Memorial Hospital, Keelung, Taiwan; ^2^Division of Colon and Rectal Surgery, Chang Gung Memorial Hospital, Keelung, Taiwan; ^3^Department of Internal Medicine, Chang Gung Memorial Hospital, Keelung, Taiwan

## Abstract

Heme oxygenase 1 (Hmox1) plays an important role in the growth and spread of tumor, and its expression is regulated positively by Nrf2 [nuclear factor (erythroid-derived 2)-like 2; NFE2L2] and negatively by kelch-like ECH-associated protein 1 (Keap1) and by BTB and CNC homology 1 (Bach1). Both Hmox1 and Nrf2 contribute to distant metastasis of cancer. The mRNA levels of Hmox1, Nrf2, Keap1, and Bach1 in the tumor and normal tissues of 84 subjects with colorectal cancer (CRC) were determined by real-time polymerase chain reaction. The tumor had lower Hmox1 but higher Bach1 mRNA levels than the normal tissue. The correlations of Hmox1 with components of the Nrf2 pathway were not significant in the tumor tissue of CRC subjects with distant metastasis. The ratio of Hmox1/Nrf2 mRNA level (by percentage) in the tumor tissue was lower in the subjects with distant metastasis (97.4% (84.4–111.1%)) than in those without (101.0% (92.7–136.5%)) and was a predictor for distant metastasis in CRC (odds ratio: 0.83; 95% confidence interval: 0.68–0.97) along with serum carcinoembryonic antigen (1.0027, 1.006–1.064). The mRNA level of Hmox1 in the tumor tissue of CRC is not correlated with that of the Nrf2 pathway molecules, and its ratio to the Nrf2 level may be useful for suggesting distant metastasis in CRC.

## 1. Introduction

Oxidative stress is an essential factor in the pathogenesis of gastrointestinal mucosal disease, including cancers [1], and may contribute to neoplastic transformation in colorectal cancer (CRC) through direct epithelial damage and genetic/epigenetic alterations [2]. Heme oxygenase 1 (Hmox1) can be induced by oxidative stress and may play a role in tumor induction, growth, or spread [3].

Hmox1 is one of the main effectors in cell responses regulated by the Nrf2 [nuclear factor (erythroid-derived 2)-like 2; NFE2L2] pathway [4], which is one of the major cellular defense mechanisms against oxidative stress [5]. Nrf2 is a “cap ‘n' collar” (CNC) basic leucine zipper transcription factor associated with its negative regulator, kelch-like ECH-associated protein 1 (Keap1), in the cytoplasm of unstressed cells, but is released from it and translocated to the nucleus under oxidative stress [6]. Once in the nucleus, Nrf2 competes with BTB (broad complex-tramtrack-bric-a-brac) and CNC homology 1 (Bach1) for binding small Maf proteins to form a heterodimer serving as a transcriptional activator that recognizes the antioxidant response element (ARE) in the promoters of Nrf2 itself, Keap1, Bach1, and many phase II detoxifying enzymes like Hmox1 [7]. Conversely, nuclear Hmox1 can bind to Nrf2 and stabilize it from glycogen synthase kinase 3*β*- (GSK3*β*-) mediated degradation [8]. Carbon monoxide induced by active Hmox1 can activate Nrf2 [9]. The molecules of the Nrf2 pathway interact with Hmox1 on the activity of promoter with ARE.

Hmox1 expression is mainly regulated by Nrf2. Hmox1 may counteract reactive oxygen species- (ROS-) mediated carcinogenesis, but its overexpression provides tumor cells with an aggressive survival advantage [10]. Hmox1, like Nrf2 [11], has a dual role in cancer. Hmox1 can stimulate angiogenesis and is prometastatic in some but not all cancers [12]. CRC with Hmox1 expression has a lower rate of lymphatic tumor invasion and better survival than that without [13]. Well-differentiated CRC seems to have more total but less nuclear Hmox1 expression than moderately/poorly differentiated CRC [14]. The role of Hmox1 and its interaction with the Nrf2 pathway in CRC remains uncertain. This study determined the mRNA levels of Nrf2, Keap1, Bach1, and Hmox1, both in the tumor and in normal tissues, to investigate their correlations in CRC.

## 2. Materials and Methods

Eighty-four consecutive subjects with a preoperative diagnosis of CRC were recruited after informed consent had been signed. Histopathologic evaluation was performed based on the diagnostic criteria of the World Health Organization [15], and all of the tumors were diagnosed as adenocarcinoma. The staging assessment was carried out according to the American Joint Committee on Cancer TNM-classification (7th edition) [16]. Clinical and pathologic characteristics were reviewed and recorded. Tumor size was defined as the product of the longitudinal and horizontal dimensions. The work was approved by the Institutional Review Board of Chang Gung Memorial Hospital.

### 2.1. Real-Time Polymerase Chain Reaction (PCR)

The tumor and normal colorectal tissue (>10 cm away from the margin of tumor) were embedded in OCT compound (Tissue-Tek, Sakura Finetek USA, Inc., Torrance, CA) within 30 minutes of surgical resection and stored at −20°C for less than 2 weeks before RNA extraction. A 2 mm^3^ portion of frozen tumor or normal colorectal tissue was minced, and the total RNA was extracted with the RNeasy Mini Kit (Qiagen, Hilden, Germany) and treated with RQ1 RNase-Free DNase (Promega, Madison, WI, USA) at 37°C for 10 minutes. The purity of RNA was determined spectrophotometrically. The cDNA was generated by reverse transcription (SuperScript III, Invitrogen, Carlsbad, CA, USA) using 1 *μ*g of total RNA. PCR amplification of glyceraldehyde-3-phosphate dehydrogenase (GAPDH) was conducted to confirm the integrity of cDNA. Real-time PCR was performed on a Bio-Rad iQ5 Real-Time PCR Detection System (Bio-Rad Laboratories, Hercules, CA, USA) with the following primers and TaqMan FAM-labeled MGB probes (Life Technologies, Carlsbad, CA, USA): Hs99999903_m1 for *β*-actin; Hs00202227_m1 for Keap1; Hs00975961_g1 for Nrf2; Hs00230917_m1 for Bach1; and Hs01110250_m1 for Hmox1. For each 0.5 mL Eppendorf tube, 12.5 *μ*L 2x FastStart Universal Probe Master (Roche), 1.25 *μ*L primer and probe mix, 9.25 *μ*L RNase-free water, and 2 *μ*L of cDNA were added to reach a total volume of 25 *μ*L. The following cycling conditions were for real-time PCR: preincubation with uracil-N-glycosylase at 50°C for 2 minutes and Ampli*Taq* Gold activation at 95°C for 10 minutes followed by 60 cycles of denaturation at 95°C for 15 seconds and annealing/extension at 60°C for 1 minute. The sizes of amplicons from real-time PCR were checked by gel electrophoresis for their deviation from those provided by the manufacturer. Duplicated cDNA samples of both the tumor and normal tissues from the same patient and the no-template control for each gene were included in the same real-time PCR experiment. Baseline and threshold values were automatically determined and the expression level of gene was evaluated through Normalized Gene Expression (ddCT) by the Bio-Rad iQ5 optical system software (version 2.1). The real-time PCR test was duplicated for each sample, and the reported data was the average of two readings (Ct number) adjusted by that of *β*-actin.

### 2.2. Statistical Analysis

Data analyses were performed by SPSS version 18 (SPSS, Inc., Chicago, Illinois, USA) and R Core Team [17]. Chi-square analysis was used to compare discrete variables. The Mann–Whitney and Kruskal–Wallis tests were undertaken to detect the difference between and among variables, respectively. The difference between paired data was detected by Wilcoxon signed ranks test. The correlations between variables were presented by Spearman's correlation coefficients. Logistic regression with stepwise selection [18] by Bayesian information criterion (BIC) was used to determine the significance of the following variables as predictors for distant metastasis in CRC: age, sex, serum CEA level, histological grade, tumor size, and mRNA level of an individual molecule or its ratio to that of Nrf2 expressed as a percentage. *p* < 0.05 was considered statistically significant.

## 3. Results

Table 1 shows the characteristics of 84 subjects with CRC. The extracted RNA of all samples had OD260/OD280 values greater than 1.8.  Table 2 lists the mRNA levels of molecules. The Bach1 mRNA level, when detectable, was higher in the tumor than in normal tissue. The Hmox1 mRNA level was higher in the tumor than in normal tissue whether or not Bach1 mRNA expression was detectable. The Nrf2 mRNA level of normal tissue was higher in the subjects with detectable Bach1 mRNA expression than in those without.  Table 3 presents the ratios of mRNA levels to Nrf2 levels in percentage. The tumor had higher Keap1/Nrf2 and Bach1/Nrf2 but lower Homx1/Nrf2 mRNA ratios compared to the normal tissue. The Hmox1/Nrf2 mRNA ratio in the tumor was lower in the subjects with distant metastasis than in those without. The mRNA levels of all molecules and the detectability of Bach1 mRNA in both the tumor and normal tissues were not different between subjects with and subjects without distant metastasis. The mRNA levels of all molecules and their ratios to the Nrf2 levels in both the tumor and normal tissues were not different between genders and among histological grades, clinical stages, or smoking statuses.

**Figure 1:**
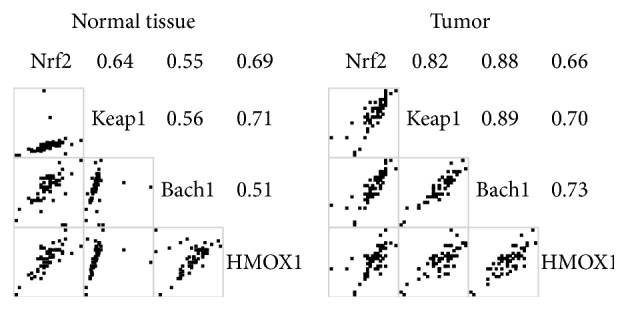
Spearman's correlation coefficients of mRNA levels in the tumor and normal tissues. All correlations are significant.

**Figure 2:**
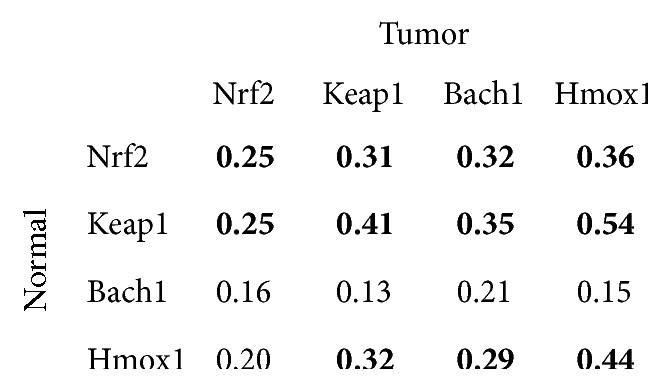
Spearman's correlation coefficients of mRNA levels between the tumor and normal tissues. A bold number denotes *p* value less than 0.05.

**Figure 3:**
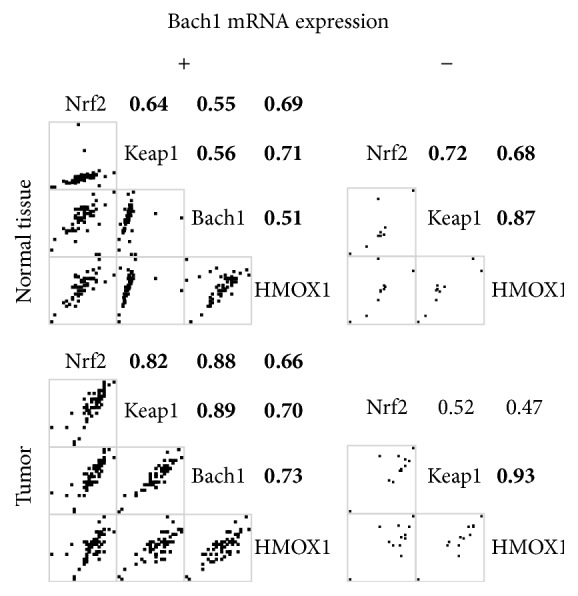
Spearman's correlation coefficients of mRNA levels in the tumor and normal tissues of subjects with or without detectable Bach1 mRNA expression. A bold number denotes a *p* value less than 0.05.

**Figure 4:**
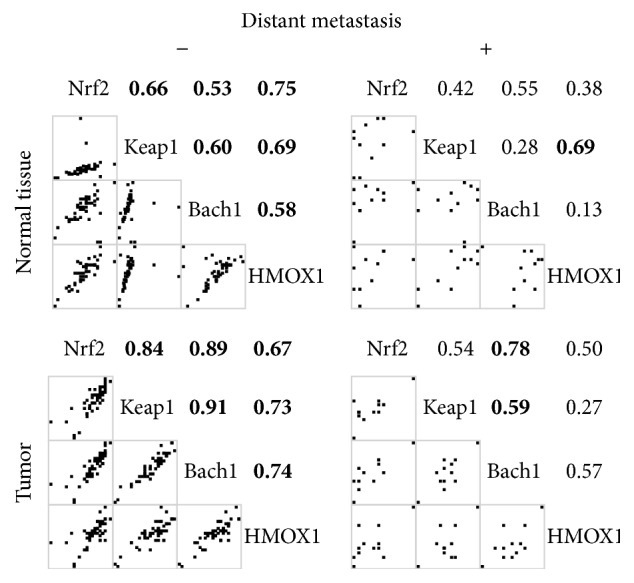
Spearman's correlation coefficients of mRNA levels in the tumor and normal tissues of subjects with or without distant metastasis. A bold number denotes a *p* value less than 0.05.

Figure 1 shows Spearman's correlation coefficients between mRNA ratios. The mRNA levels were all significantly correlated in the tumor and normal tissue.  Figure 2 presents the correlations of mRNA levels between the tumor and normal tissues, which were significant in Nrf2, Keap1, and Hmox1, but not in Bach1.  Figure 3 shows the correlations of mRNA levels in the normal and tumor tissues with or without detectable Bach1 mRNA expression. The Nrf2 mRNA level lost significant correlations with those of Keap1 and Hmox1 in the tumor tissue of subjects without detectable Bach1 mRNA expression.  Figure 4 presents the correlations of mRNA levels in the normal and tumor tissues with and without distant metastasis. In subjects with distant metastasis, the correlations between the mRNA levels of Nrf2 and Hmox1 were not significant in either the tumor or normal tissues.

The logistic regression with stepwise selection by BIC found that the ratio of Hmox1/Nrf2 mRNA by percentage in the tumor tissue (odds ratio: 0.83; 95% confidence interval: 0.68–0.97) and CEA (1.0027; 1.006–1.064) were predictors for distant metastasis in CRC.

## 4. Discussion

The tumor had lower Hmox1 but higher Bach1 mRNA levels and ratio with Nrf2 than the normal tissue in CRC. In the absence of detectable Bach1 mRNA expression, the mRNA levels of Keap1, but not Nrf2, retained significant correlation with that of Hmox1 in the tumor tissue of CRC. The significant correlations between the mRNA levels of Hmox1 and Nrf2 were lost in both the tumor and normal tissues of CRC subjects with distant metastasis. The Hmox1/Nrf2 mRNA ratio in the tumor tissue was lower in CRC subjects with distant metastasis than in those without and was a significant predictor of distant metastasis in CRC.

The development of both sporadic and colitis-associated CRC involves many of the same genetic defects, which may be caused by oxidative stress [2]. Nrf2 is the principal transcription factor affecting the expression of phase II antioxidant enzymes like Hmox1 [19]. Nrf2 can be constitutively overexpressed in cancer cells or tumor tissues and is a protooncogene that can suppress or promote tumor [10, 20]. Hmox1 can protect tissue against oxidative stress [21] and is a key effector of Nrf2 upregulation in tumor progression [4]. The Nrf2/Hmox1 axis is a double-edged sword in cancer [4]. Many molecules, such as Keap1, Fyn, and Bach1, whose transcription is regulated by Nrf2, can increase the degradation of Nrf2 or compete with its binding to ARE on the promoter, while other molecules like sequestosome 1 and PALB2 can stabilize Nrf2 [22]. Under oxidative stress, nuclear translocated Hmox1 interacts with Nrf2 and protects it from GSK3*β*-mediated phosphorylation coupled with ubiquitin-proteasomal degradation, thereby prolonging its accumulation in the nucleus [8]. The function of Nrf2 as a transcription factor is affected by many proteins and autoregulatory loops [23].

Although epigenetic modification affects the expression of Nrf2 and Keap1 in many ways [24], their mRNA levels are not different between the tumor and normal tissues. The tumor had lower Hmox1 and higher Bach1 mRNA levels than the normal tissue. Hmox1 had mRNA expression lower in the tumor than in the normal tissue by gene array [25]. Bach1 plays a critical role in the negative regulation of Hmox1 transcription through the StRE- (stress-responsive element-) Bach1-Nrf2 axis [21]. The Bach1 mRNA level is regulated by many factors like Nrf2, heme, Raf kinase inhibitor protein (RKIP), miR-155-5p, and itself [7, 26–28]. Bach1 mRNA expressions were undetectable in some subjects (22/84) and were insignificantly correlated between the tumor and normal tissues, suggesting that the regulation of Bach1 expression is different between tissues and among subjects of CRC. In the tumor tissue with undetectable Bach1 mRNA expression, the correlation between the mRNA levels of Keap1 and Hmox1 was the only one that remained significant. In keratinocytes, knockdown of Bach1, Keap1, and Nrf2 has fold changes of Hmox1 mRNA transcription of 136.4, 2.3, and 0.4, respectively [29]. Without Bach1 expression, Keap1 may become the major regulator of Hmox1 mRNA transcription in CRC tumor tissue. Bach1 may play a role in the transcriptional regulation of Hmox1 by the Nrf2-Keap1 pathway in the tumorous CRC tissue, but not the normal tissue.

Hmox-1 may increase the metastatic potential of cancer due to its proangiogenic property [3]. However, Hmox-1 expression in CRC is associated with prolonged survival and a low rate of lymphatic tumor invasion [13]. Inhibition of Hmox1 can increase the liver metastasis of CRC in mice [30]. The role of Hmox1 in tumor metastasis remains unclear [12]. Bach1 promotes the liver metastasis of CRC by upregulating c-Myc and SOX4 [31]. RKIP, a downregulator of Bach1 expression [27], is reduced in the metastatic tumor of CRC [32]. High level of miR-155-5p, which can target Bach1 mRNA [28], in the tumor tissue of CRC is associated with increased lymph node metastasis [33]. The role of Bach1 in the metastasis of CRC is uncertain. The role of Nrf2 in tumor metastasis remains to be clarified [10]. Frequent hypermethylation of Keap1 promoter reduces its mRNA transcription but is not associated with clinicopathological features in CRC [34]. The mRNA levels of Hmox1 and molecules of the Nrf2 pathway in either the tumor or normal tissues were not predictors for distant metastasis in CRC.

Hmox1 has many regulatory domains other than StRE on its promoter and its mRNA transcription is affected by many factors like NF-*κ*B, IL-6, and STAT3 [21]. Conversely, the transcription of Nrf2 can be regulated by factors other than itself [23, 24] and increased by K-Ras, B-Raf, and Myc in tumor cells [22]. Epigenetic alterations by miRNAs upregulate Bach1 and downregulate Keap1, K-ras, STAT3, and Myc expression in CRC [28]. ROS can regulate the activity of Nrf2 through redox factor-1 [35]. Variable ratios of Hmox1/Nrf2 mRNA levels may represent that the transcription regulations of Hmox1 and Nrf2 are modified by factors other than Nrf2, Keap1, and Bach1. A disease is a consequence of the complex intracellular network rather than a single gene [36] and so is cancer metastasis [37]. Although the Nrf2/Hmox1 axis is important in tumor progression [4], Nrf2 and Hmox1 have uncertain roles in cancer metastasis [10, 12]. Many factors involving the transcriptional regulation of Nrf2 and Hmox1 play roles in the metastasis of CRC [10, 25, 31, 38–40]. The lack of significant correlations between Nrf2 and Hmox1 mRNA levels in both the tumor and normal tissues suggests that the transcriptional regulation of Hmox1 by Nrf2 is loose in CRC subjects with distant metastasis. The ratio of Hmox1/Nrf2 mRNA levels in the tumor tissue may reflect a difference in their transcriptional regulation and was a negative predictor for distant metastasis in CRC.

ROS plays an important role in the carcinogenesis of CRC [2] and metastasis in cancer [35]. Nrf2 and Hmox1 can protect cells from oxidative stress [4] and play roles in tumor metastasis [4, 10]. The transcriptional regulations of Nrf2 and Hmox1 are complex and modified by many factors [21–24, 28, 35]. The Hmox1/Nrf2 mRNA ratio in the tumor tissue may be a useful indicator for distant metastasis in CRC. The differential expressions of Nrf2 and Hmox1 mRNA and their relation to distant metastasis in CRC warrant further investigation.

## Figures and Tables

**Table 1:** Characteristics of 84 subjects with colorectal cancer.

Age (years)	68.3 ± 13.0
Gender (male/female)	48 (57%)/36 (43%)
CEA	2.02 (0.5–1326.1)
Clinical stage	
I	11 (13%)
II	26 (31%)
III	33 (39%)
IV	14 (17%)
Histological differentiation	
Well	18 (21%)
Moderate	56 (67%)
Poorly	10 (12%)
Tumor size, cm	26.3 ± 19.7

**Table 2:** mRNA levels in the tumor and normal tissues of colorectal cancer with or without detectable Bach1 mRNA.

	Bach1 mRNA detectable^†^		Bach1 mRNA not detectable^‡^	
	*n* = 62		*n* = 22	
	Normal tissue	Tumor	Normal tissue	Tumor
Nrf2	0.93 (0.82–1.03)	0.92 (0.75–1.00)	0.95 (0.68–1.42)^*∗∗*^	0.90 (0.52–1.13)
Keap1	1.00 (0.85–1.81)	1.01 (0.86–1.08)	0.98 (0.83–1.36)	0.99 (0.74–1.49)
Hmox1	0.98 (0.76–1.12)^*∗*^	0.92 (0.78–1.03)	1.01 (0.74–1.48)^‡^	0.88 (0.56–1.10)
Bach1	0.93 (0.75–1.04)^*∗*^	0.94 (0.80–1.03)	—	—

^†^Subjects with Bach1 mRNA expression detectable in both the tumor and normal tissues.

^‡^Subjects with Bach1 mRNA expression not detectable in the tumor or normal tissue.

^*∗*^Significantly different from the tumor tissue by Wilcoxon signed ranks test.

^*∗∗*^Significantly different from subjects with detectable Bach1 mRNA expression by Mann–Whitney test.

**Table 3:** Ratios of mRNA levels to that of Nrf2 by percentage in colorectal cancer.

%		Keap1/Nrf2	Bach1/Nrf2	Hmox1/Nrf2
Normal tissue		107.1 (88.8–198.9)^*∗*^	100.0 (76.5–107.2)^*∗*^	105.1 (92.7–137.0)^*∗*^
Tumor		109.6 (102.3–146.0)	102.2 (95.2–116.0)	100.0 (84.4–136.5)
	Distant metastasis			
Normal tissue	With	108.6 (101.0–140.2)	100.0 (90.0–106.7)	106.3 (96.7–137.0)
	Without	106.9 (88.8–198.9)	100.0 (76.5–107.3)	104.3 (92.7–135.8)
Tumor	With	109.1 (105.0–116.0)	102.2 (98.0–106.0)	97.4 (84.4–111.1)^*∗∗*^
	Without	109.6 (102.0–146.0)	102.2 (95.0–116.0)	101.0 (92.7–136.5)

^*∗*^Significantly different between the tumor and normal tissues by Wilcoxon signed ranks test.

^*∗∗*^Significantly different between tissues of subjects with or without distant metastasis by Mann–Whitney test.
